# Comparison of ActiGraph GT9X Link with two Japanese accelerometers for assessments of free-living physical activity in junior high school students

**DOI:** 10.1186/s13104-020-05231-x

**Published:** 2020-08-20

**Authors:** Kensaku Sasayama, Minoru Adachi

**Affiliations:** 1 Faculty of Education, Okayama University of Science, 1-1, Ridai-cho, Kita-ku, Okayama, 700-0005 Japan; 2 Graduate School of Education, Okayama University, 3-1-1, Tsushima-naka, Kita-ku, Okayama, 700-8530 Japan

**Keywords:** ActiGraph, Lifecorder, Active style pro, Total steps, Moderate-to-vigorous physical activity

## Abstract

**Objective:**

This study compared the measurements of total steps and moderate-to-vigorous physical activity (MVPA) between ActiGraph and the Lifecorder and Active Style Pro accelerometers in junior high school students.

**Results:**

The total steps and MVPA significantly differed between ActiGraph and Lifecorder measurements, whereas the intraclass correlation coefficients (ICCs) for total steps and MVPA between the two accelerometers were not significant. There was no significant difference between the total steps measured using ActiGraph and Active Style Pro, and moderate agreement was noted. Additionally, MVPA measured using ActiGraph and Active Style Pro significantly differed, whereas the ICC for MVPA measured using the two accelerometers was not significant. When comparing the total steps between ActiGraph and Lifecorder and MVPA between ActiGraph and Lifecorder or Active Style Pro, it should be noted that the accelerometer measurements are not compatible.

## Introduction

Physical activity during youth has a positive effect on mental and physical health [1, 2]. Therefore, an indicator of physical activity in the World Health Organization guidelines is ≥ 60 min of moderate-to-vigorous physical activity (MVPA) per day [3]. Total steps have been assessed as an objective and comprehensible indicator of physical activity, and studies on total steps equivalent to 60 min of MVPA per day have been reported [4, 5].

Total steps and MVPA can be objectively measured using an accelerometer. ActiGraph (ActiGraph Inc., Florida, USA) has been internationally used to objectively evaluate physical activity [6, 7]. Although ActiGraph has several standardized MVPA evaluation methods, differences in MVPA evaluated using various cutoffs and epoch length have been reported [8, 9]. Therefore, previous studies have examined the compatibility of MVPA evaluated using ActiGraph [10].

In Japan, Lifecorder (Suzuken Co. Ltd., Nagoya, Japan) [11, 12] and Active Style Pro (Omron Healthcare Co. Ltd., Kyoto, Japan) [13, 14] are frequently used to evaluate physical activity. However, studies of the compatibility of the results of ActiGraph, which is frequently used internationally, with those of Lifecorder and Active Style Pro are insufficient. To our knowledge, only McClain et al. [15] compared ActiGraph and Lifecorder in 10-year-old children. Total steps and MVPA calculated using ActiGraph, Lifecorder, and Active Style Pro have not been compared in junior high school students. By examining the compatibility of physical activity evaluated using these devices, previous findings can be further utilized. Therefore, we compared total steps and MVPA calculated using ActiGraph, Lifecorder, and Active Style Pro accelerometers.

## Main text

### Methods

#### Participants

This study was conducted in 2017 at a junior high school (Okayama, Japan) with 20 male junior high school students (mean age, 12.3 ± 0.5 years). Their mean height, weight, and body mass index were 149.5 ± 8.2 cm, 43.0 ± 10.3 kg, and 19.0 ± 3.2 kg/m^2^, respectively. The Institutional Review Board of the Okayama University of Science approved this study (No. 29-4). All participating children and their parents provided written informed consent before participation.

#### Physical activity

To assess total steps and MVPA, the ActiGraph GT9X Link (AG), Lifecorder EX 4 sec version (LC), and Active Style Pro HJA-750C (ASP) accelerometers were used. Participants wore AG and LC or ASP (10 each) on their right waists from 8:30 a.m. to 4:30 p.m. on one weekday. Data of 7 h, i.e., from 9:00 a.m. to 4:00 p.m., was used for analysis.

The validity of AG for assessing physical activity in children has been confirmed, and this accelerometer is widely used internationally [16]. However, there is no international consensus regarding the cutoff and epoch length used to calculate MVPA for children using AG [8, 9]. Our cutoff value of MVPA is same as that used by Puyau [17], which was reported to be used relatively frequently by Cain et al. [8]. Shorter epoch settings are recommended for children [18]. However, in our study, the epoch length was 60 s because this value was used in 62.3% of studies conducted with children (aged 2–18 years) published between 2005 and 2010 [8].

LC (72.5 × 41.5 × 27.5 mm, 60 g including the battery) is an uniaxial accelerometer commonly used in Japan. Kumahara et al. [19] reported that this accelerometer samples acceleration at a rate of 32 samples/s and assesses values ranging from 0.06 to 1.94 g. Acceleration signal was filtered using an analog bandpass filter and subsequently digitized. The maximum pulse over 4 s was measured as acceleration value and classified into 11 activity levels (0, 0.5, and 1–9). Adachi et al. [20] reported that the total energy expenditure assessed using the doubly labeled water method was significantly correlated with total steps and activity levels (LC1–6 and LC7–9) detected using LC in junior high school students (total steps, r = 0.79, p < 0.05; LC1–6, r = 0.71, p < 0.05; LC7–9, r = 0.83, p < 0.05). Additionally, Sasayama and Adachi [21] found that the activity level measured using LC was significantly correlated with metabolic equivalents (METs) during walking and running in junior high school students (r = 0.883, p < 0.05). They confirmed that the activity level for MVPA (≥ 3 METs) is equivalent to a value of ≥ 5 as detected using LC. Therefore, the MVPA cutoff value was based on these findings [21].

Similar to LC, Active Style Pro HJA-350IT (76 × 46 × 34 mm, 60 g including the battery) is a triaxial acceleromter commonly used in Japan. Ohkawara et al. [22] reported that this accelerometer samples acceleration at a rate of 32 samples/s and assesses values ranging from − 6 to 6 g. The epoch length of ASP is 10 s. Ohkawara et al. [22] reported a formula for estimating MVPA from acceleration obtained in adults. In addition, Hikihara et al. [23] reported an equation for estimating MVPA from acceleration obtained in children aged 6–12 years. We used ASP (40 × 52 × 12, 23 g including the battery) miniaturized by the same algorithm as Active Style Pro HJA-350IT, and MVPA was calculated using the estimation equation described by Hikihara et al. [23].

#### Statistical analysis

Differences of physical activity as measured using AG versus LC or ASP were analyzed using paired Student’s t-test. Agreement of physical activity between AG and LC or ASP was analyzed using intraclass correlation coefficient (ICC). ICC of < 0.40 indicated poor agreement, ICC of 0.40–0.75 indicated moderate agreement, and ICC of > 0.75 indicated excellent agreement [24]. Bland–Altman plots were analyzed to investigate differences between the accelerometer measurements. All analyses were performed using IBM SPSS Statistics software version 24 (IBM Japan, Ltd., Tokyo, Japan). Results were considered statistically significant at p-value of < 0.05.

### Results

#### AG and LC

Table 1 presents the ICCs for total steps and MVPA measured using AG and LC. For total steps and MVPA, the ICCs between AG and LC were − 0.20 (p = 0.72) and − 0.19 (p = 0.72), respectively. Total steps (p = 0.004) and MVPA (p = 0.020) were both significantly higher for the LC measurements than for the AG measurements.

**Table 1:** ICCs for total steps and MVPA measured using AG and LC

	AG	LC	ICC	95% CI	p values
Total steps (steps)	2176.6 (506.0)	4994.2 (2588.4)*	−0.20	−0.70–0.45	0.72
MVPA (min)	3.3 (4.2)	11.0 (7.8)*	−0.19	−0.70–0.46	0.72

*MVPA* moderate-to-vigorous physical activity, *AG* ActiGraph GT9X , ink, *LC* Lifecorder EX, *ICC* intraclass correlation coefficient, *CI* confidence Interval

*p < 0.05 for differences between AG and LC

#### AG and ASP

Table 2 presents the ICCs for total steps and MVPA measured using AG and ASP. For total steps and MVPA, ICCs between AG and ASP were 0.74 (p < 0.01) and − 0.07 (p = 0.58), respectively. Although total steps did not significantly differ between AG and ASP measurements (p = 0.169), MVPA measured using ASP was significantly higher than that measured using AG (p = 0.002).

**Table 2:** ICCs for total steps and MVPA measured using AG and ASP

	AG	ASP	ICC	95% CI	p values
Total steps (steps)	2241.4 (896.8)	2597.9 (1266.7)	0.74	0.28–0.93	< 0.01
MVPA (minutes)	2.9 (3.4)	13.8 (9.7)*	−0.07	−0.62–0.55	0.58

*MVPA* moderate-to-vigorous physical activity, *AG* ActiGraph GT9X Link, *ASP* Active Style Pro HJA-750C, *ICC* intraclass correlation coefficient, *CI* confidence interval

*p < 0.05 for differences between AG and ASP

#### Bland–Altman plots of AG, LC and ASP

**Fig. 1:**
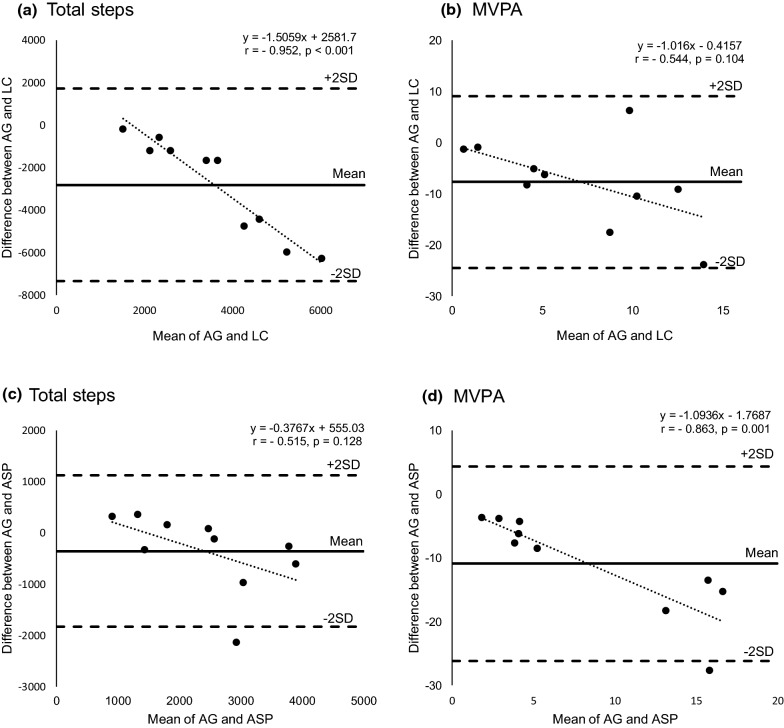
Bland–Altman plots for AG, LC, and ASP. **a** AG and LC mean total steps, **b** AG and LC mean MVPA, **c** AG and ASP mean total steps, **d** AG and ASP mean MVPA

The Bland–Altman plots presented in Fig. 1 show the degree of differences in total steps and MVPA among AG, LC, and ASP measurements. Between AG and LC, ICCs between the mean of the measures and bias were − 0.95 (p < 0.001) for total steps and − 0.54 (p = 0.10) for MVPA.

Between AG and ASP, the ICCs between the mean of the measures and bias were − 0.52 (p = 0.13) for total steps and − 0.86 (p = 0.001) for MVPA.

### Discussion

This study compared measurements of total steps and MVPA between AG and the LC and ASP accelerometers in junior high school students. Total steps and MVPA differed between AG and LC. We also found that MVPA differs between AG and ASP. Total steps and MVPA among AG, LC, and ASP showed that only MVPA between AG and ASP was in moderate agreement.

Total number of steps was significantly lower when measured using AG than when measured using LC; however, the ICC of total steps as measured using AG and LC indicated poor agreement. MVPA as measured using AG was also significantly lower than that measured using LC, whereas the ICC of MVPA measured using AG and LC indicated poor agreement. McClain et al. [15] reported that total steps as measured using AG (11,770 ± 2529 steps/day) were significantly higher than those measured using LC (10,969 ± 2446 steps/day). To explain the discrepancy, the researchers suggested that LC is less affected by random nonambulatory vibrations or postural changes than AG. However, our findings were not consistent with McClain’s results. Although the study was conducted in adults, the results of comparison of the ActiGraph and Lifecorder step counts were not consistent with the reports of higher ActiGraph [25] and higher Lifecorder [26]. Abel et al. [26] have reported that these discrepancies may be owing to the differences in ActiGraph models. The difference between the present study and McClain’s study (ActiGraph GT1M, Lifecorder EX) may be owing to the different models of ActiGraph and Lifecorder. The cause of the discrepancy between the McClain et al. [15] and our study is unclear and requires additional investigation; however, both studies indicate that the direct comparison of total steps between ActiGraph and Lifecorder should be avoided.

Regarding MVPA measurements, McClain et al. [15] reported no significant difference in the comparison of MVPA between AG (27.3 ± 15.4 min/day) and LC (25.9 ± 9.4 min/day) using the cutoff values for MVPA identified by Puyau et al. [17] and the cutoff value of ≥ LC5 for LC as used in our study. Although MVPA measurements significantly differed between AG (3.3 ± 4.2 min/day) and LC (11.0 ± 7.8 min/day), this was attributed to differences in the epoch length. To date, previous studies reported that MVPA measurements vary depending on epoch length [18, 27]. McClain et al. [15] used an epoch length of 15 s for AG, whereas our study used an epoch length of 60 s for LC, possibly explaining the difference in MVPA measurements between AG and LC. Similar to McClain et al. [15], we analyzed 15-s epoch and there was no significant difference between AG and LC (AG; 6.7 ± 7.5 min/day, p = 0.25, data not shown in table). Therefore, even if the same MVPA cutoff value is used, AG and LC will provide different data for MVPA if epoch length is different.

To our knowledge, no study compared the results of total steps and MVPA between AG and ASP in junior high school students. Our results revealed no significant difference in total steps between AG and ASP measurements. Although the study was conducted in adults, Yano et al. [28] examined step counts between ActiGraph GT3X and Active Style Pro HJA-350IT and reported that step counts in Active Style Pro HJA-350IT were higher than those in ActiGraph GT3X. Additionally, Yano et al. [28] reported that the measurement difference between Active Style Pro HJA-350IT and ActiGraph GT3X tended to be higher when mean measurement between Active Style Pro HJA-350IT and ActiGraph GT3X was larger. The absence of difference between AG and ASP in our study may be owing to the short measurement time (7 h/day). For comparisons of MVPA measurements between AG and ASP, MVPA measured using AG was significantly lower than that measured using ASP, whereas the ICC for MVPA as measured using AG and ASP indicated poor agreement. The Bland–Altman plot for AG and ASP measurements of MVPA revealed proportional bias between the accelerometers. Caution should be taken when comparing MVPA between AG and ASP.

### Conclusions

Total steps and MVPA as evaluated using AG were lower than those obtained using LC, and the discrepancy was larger for higher values. Additionally, moderate agreement was identified for total steps evaluated using AG and ASP. Conversely, MVPA evaluated using AG was lower than that evaluated using ASP, and the discrepancy was larger for higher values. Thus, when comparing the total steps between AG and LC and MVPA between AG and LC or ASP, it should be noted that the accelerometer measurements are not compatible.

## Limitations

MVPA value as measured using AG differs according to the selected cutoff value and epoch length [18, 27]. Therefore, our results may differ depending on the selected cutoff value and epoch length. Although physical activity was assessed in the free-living setting, the difference in physical activity among the devices may be larger if a whole-day assessment is performed because activities before and after school were not included.

## Data Availability

Please contact the corresponding author for data requests.

## Abbreviations

MVPA: Moderate-to-vigorous physical activity
AG: ActiGraph GT9X Link
LC: Lifecorder EX 4 sec
ASP: Active Style Pro HJA-750C
MET: Metabolic equivalent
ICC: Intraclass correlation coefficient
